# Mitochondrial Supercomplexes Do Not Enhance Catalysis by Quinone Channeling

**DOI:** 10.1016/j.cmet.2018.05.024

**Published:** 2018-09-04

**Authors:** Justin G. Fedor, Judy Hirst

**Affiliations:** 1 The Medical Research Council Mitochondrial Biology Unit, University of Cambridge, Wellcome Trust/MRC Building, Cambridge Biomedical Campus, Hills Road, Cambridge CB2 0XY, UK

**Keywords:** alternative oxidase, channeling, mitochondria, oxidative phosphorylation, respirasome, supercomplex, ubiquinone

## Abstract

Mitochondrial respiratory supercomplexes, comprising complexes I, III, and IV, are the minimal functional units of the electron transport chain. Assembling the individual complexes into supercomplexes may stabilize them, provide greater spatiotemporal control of respiration, or, controversially, confer kinetic advantages through the sequestration of local quinone and cytochrome *c* pools (substrate channeling). Here, we have incorporated an alternative quinol oxidase (AOX) into mammalian heart mitochondrial membranes to introduce a competing pathway for quinol oxidation and test for channeling. AOX substantially increases the rate of NADH oxidation by O_2_ without affecting the membrane integrity, the supercomplexes, or NADH-linked oxidative phosphorylation. Therefore, the quinol generated in supercomplexes by complex I is reoxidized more rapidly outside the supercomplex by AOX than inside the supercomplex by complex III. Our results demonstrate that quinone and quinol diffuse freely in and out of supercomplexes: substrate channeling does not occur and is not required to support respiration.

## Highlights

• AOX can be added *in vitro* to membranes to catalyze quinol oxidation
• Respiratory supercomplexes do not speed up electron transfer by channeling quinone
• AOX addition can probe respiratory-chain function using endogenous quinones

## Graphical Abstract

**Figure:**
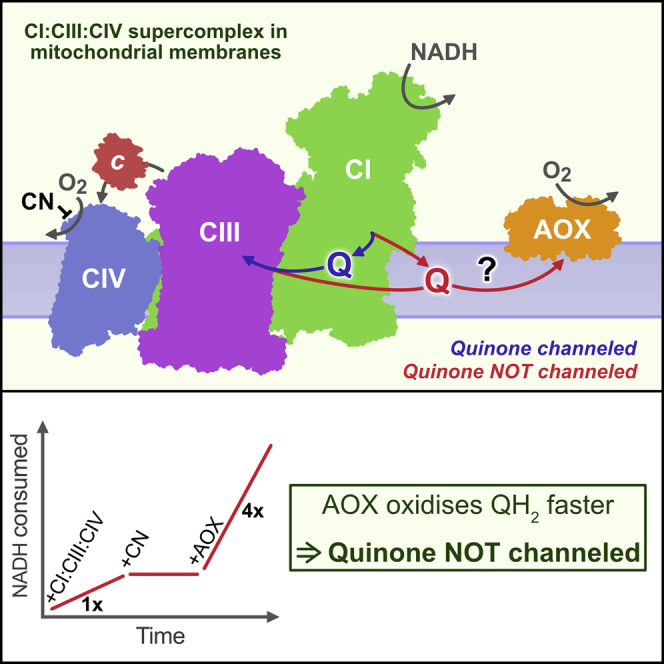

## Teaser

Mitochondrial respiratory supercomplexes catalyze aerobic respiration. One of the reasons proposed for their supramolecular organization is to facilitate rapid electron transfer between complexes I and III via quinone channeling. Fedor et al. demonstrate that this is not the case, and that quinone functions as a shared, freely exchanging pool in mitochondria.

## Introduction

Mitochondrial respiration, catalyzed predominantly by supermolecular assemblies of respiratory complex I (CI, NADH:ubiquinone oxidoreductase), complex III (CIII, ubiquinol:cytochrome *c* oxidoreductase), and complex IV (CIV, cytochrome *c* oxidase), is at the center of cellular bioenergetics (Lenaz et al., 2016, Letts and Sazanov, 2017, Lobo-Jarne and Ugalde, 2018, Milenkovic et al., 2017). The CI_1_:CIII_2_:CIV_1_ “respirasome” is generally considered the minimal functional unit of the respiratory chain, and its formation has been proposed to stabilize CI through close interactions with CIII and CIV, and to mitigate the production of reactive oxygen species (ROS) (Letts et al., 2016, Lobo-Jarne and Ugalde, 2018, Maranzana et al., 2013). More controversially, the close association of the enzymes in supercomplex assemblies has been suggested to confer a kinetic advantage on respiration by trapping or channeling quinone to enhance its transfer between the enzymes in the supercomplex, creating an independent, local quinone pool that does not exchange with the quinone pool outside (reviewed in Lenaz et al., 2016, Milenkovic et al., 2017).

Quinone channeling between the respiratory complexes was proposed originally on the basis of flux control analyses (Bianchi et al., 2004), then supported by genetics-based studies that suggested mitochondrial supercomplexes sequester their own dedicated quinone/quinol pools (Lapuente-Brun et al., 2013). An even larger assembly than the respirasome, the so-called megacomplex, CI_2_:CII_2_:CIII_2_:CIV_2_, was recently proposed and suggested to contain a sealed-in quinone pool for maximum catalytic effectiveness (Guo et al., 2017). In a less restrictive model it has also been proposed that, although exchange with the outside is not precluded, under high turnover conditions quinone/quinol react preferentially within the supercomplex (Lenaz et al., 2016, Letts et al., 2016) due to the enzyme proximities. Alternatively, recent structures of the mammalian respirasome clearly show that the substrate-binding sites in CI and CIII are separated by ∼100 Å, with no mediating protein to facilitate channeling between them (Gu et al., 2016, Guo et al., 2017, Letts et al., 2016, Milenkovic et al., 2017, Wu et al., 2016). Similarly, the structures reveal no barriers to the free diffusion of cytochrome *c* (Letts et al., 2016, Milenkovic et al., 2017), which has been shown by biophysical methods to diffuse freely along the membrane and not be localized to a single supercomplex (Trouillard et al., 2011). Finally, kinetic experiments have shown that CI and complex II (CII, succinate:ubiquinone oxidoreductase) are both able to reduce all the CIII present, suggesting they do not interact with separate quinone pools (Blaza et al., 2014).

**Figure 1:**
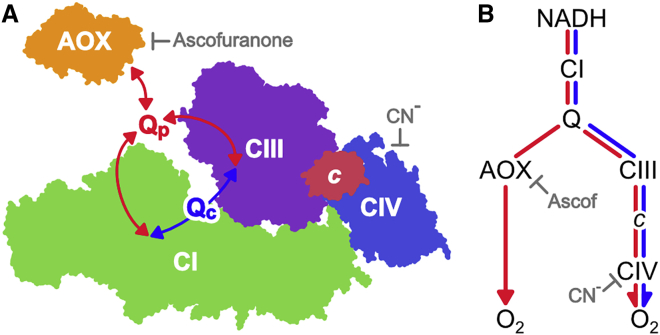
Pictorial Representation of Quinone/Quinol Cycling during NADH Oxidation by a Respiratory-Chain Supercomplex in the Presence of AOX (A) The CI_1_:CIII_2_:CIV_1_ supercomplex might confine channeled quinone/quinol (Q_C_, blue pathway) to shuttle between CI and CIII within the supercomplex assembly, or depend on quinone/quinol exchange with the quinone pool (Q_P_, red pathway). The competing quinol oxidase (AOX) can only react with quinol from the pool. Image created from the structures of the ovine respirasome (Letts et al., 2016) and *T. brucei* AOX (Shiba et al., 2013). (B) Flow diagram showing flux through the CIII/CIV and AOX pathways if quinone is channeled (blue pathway) or behaves as a shared pool (red pathways).

Introduction of an external enzyme to compete for a potentially channeled substrate is a diagnostic test for channeling (Bulutoglu et al., 2016, Wheeldon et al., 2016). If the substrate is truly channeled, flux through the competing pathway is negligible. The alternative oxidase (AOX) from *Trypanosoma brucei brucei* is a cyanide-insensitive quinol oxidase that does not contribute to the proton motive force (Δ*p*) (Shiba et al., 2013). Here, we describe how AOX can be added *in vitro* to preparations of the inner membrane of mammalian mitochondria, providing an additional pathway for ubiquinol oxidation that competes with the CIII/CIV pathway. Addition of AOX thereby provides a simple strategy for testing whether ubiquinone is channeled in mitochondrial supercomplexes. Figure 1 illustrates how, in the presence of AOX, channeled quinone (Q_C_) may continue to provide a substantial flux of electrons through the supercomplex (CI_1_:CIII_2_:CIV_1_), in which almost all the CI is incorporated (Lobo-Jarne and Ugalde, 2018, Schägger, 2000). Because it includes CIV, the supercomplex flux is sensitive to inhibition by cyanide. Alternatively, quinone that behaves as a pool (Q_P_), exchanging in and out of the supercomplex, also supports flux through the AOX pathway. The AOX flux is sensitive to inhibition by ascofuranone, but not cyanide (Minagawa et al., 1996).

Here, we use coupled submitochondrial particles (SMPs), inverted sealed vesicles of the inner membrane from bovine heart mitochondria, to reveal that addition of AOX substantially boosts the rate of NADH:O_2_ oxidoreduction and renders it insensitive to inhibition by cyanide. Therefore, AOX competes effectively with the CIII/CIV pathway. We take advantage of the well-established SMP system to demonstrate that neither the membrane integrity nor the supercomplex assemblies are perturbed by AOX, and that the respiratory enzymes remain fully competent for oxidative phosphorylation. Therefore, quinone is not channeled or sequestered by the respiratory-chain supercomplexes: it operates as a common pool, shared between all enzymes, and supercomplex assemblies do not trap quinone/quinol molecules between CI and CIII in order to enhance catalysis.

## Results

### Addition of AOX to Mammalian Respiratory Membranes Shows that Quinone/Quinol Is Not Channeled

**Figure 2:**
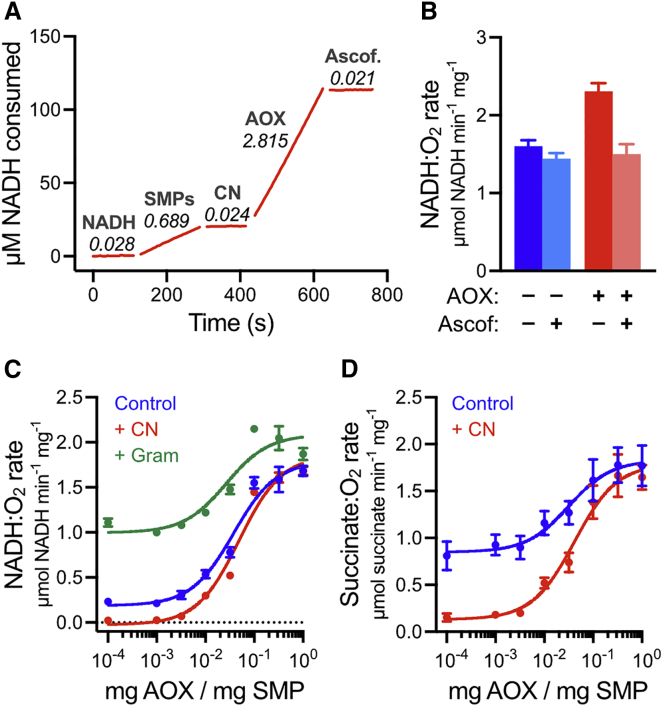
Addition of AOX Stimulates Catalysis by SMPs from Bovine Heart Mitochondria (A) Kinetic assay trace showing how catalysis responds to addition of 0.1 mg AOX per mg SMP. Rates of catalysis are marked in μmol NADH min^−1^ mg^−1^. A total of 200 μM NADH, 10 μg mL^−1^ SMPs, 400 μM NaCN, 0.1 mg AOX mg^−1^, and 500 nM ascofuranone were added sequentially as indicated. (B) Rates of NADH oxidation in SMPs, uncoupled using 1 μg mL^−1^ gramicidin and treated with 0.1 mg AOX mg^−1^ and/or 300 nM ascofuranone. DMSO (the vehicle for ascofuranone) was added to ascofuranone-free experiments at 0.1%. All values are mean ± SD (n = 6). (C and D) The effects of supplementing SMPs with increasing concentrations of AOX on the rates of succinate:O_2_ (C) and NADH:O_2_ (D) oxidoreduction. Conditions: 10 μg mL^−1^ SMPs, 200 μM NADH, 400 μM NaCN, 1 μg mL^−1^ gramicidin. Control: 0.2% DMSO (the vehicle for gramicidin). All values are mean ± SD (n = 6).

If respiratory-chain supercomplex assemblies contain the quinone/quinol substrate and channel it between their component enzymes, then a competing quinol oxidase, outside the supercomplex structure, should not be able to turn over (Bulutoglu et al., 2016, Wheeldon et al., 2016) (Figure 1). To test the effects of adding a competing quinol oxidase to mammalian respiratory membranes, we added 0.1 mg AOX per mg SMPs (hereafter written as 0.1 mg AOX mg^−1^) directly to a suspension of SMPs while monitoring the rate of NADH oxidation (NADH:O_2_ oxidoreduction). Figure 2A reveals that AOX binds to the SMPs and catalyzes quinol oxidation immediately. The same effect was also observed in synthetic, CI-only proteoliposomes containing ubiquinone-10, and in mitochondrial membrane fragments. AOX-catalyzed quinol oxidation is insensitive to cyanide, which inhibits CIV and thus CIII/CIV-catalyzed quinol oxidation, but is sensitive to ascofuranone (Minagawa et al., 1996). Crucially, in SMPs the cyanide-insensitive rate of quinol oxidation by AOX, which by definition cannot involve quinone/quinol channeling, is up to four times greater than the cyanide-sensitive rate of quinol oxidation by CIII/CIV, despite the SMPs being supplemented with additional cytochrome *c* to support CIII/CIV catalysis. Figure 2B, recorded using the uncoupler gramicidin to collapse Δ*p*, confirms that the increased rate is due specifically to AOX, as the rates from ascofuranone-treated SMPs and AOX-SMPs are not significantly different (two-tailed t test, p value 0.36).

To further explore the 4-fold higher rate of NADH oxidation observed through the AOX pathway than the CIII/CIV pathway in Figure 2A, we performed AOX titrations on both NADH:O_2_ and succinate:O_2_ oxidoreduction (Figures 2C and 2D). For both reactions, the rate increases to a plateau at ∼0.1 mg AOX mg^−1^. As shown in the presence of cyanide, the CIII/CIV pathway dominates at low AOX levels and the AOX pathway at high AOX levels. Finally, to exclude the possibility that the increase arises only from the lower H^+^/2*e*^−^ stoichiometry of the AOX pathway (i.e., with fewer protons to force against Δ*p*, AOX-supported catalysis is less hindered), NADH oxidation data were recorded in the presence of gramicidin to collapse Δ*p*. Figures 2C and 2B confirm that AOX still causes a substantial increase in the rate of catalysis. Therefore, in the absence of AOX, CIII/CIV catalysis is rate limiting for both NADH and succinate oxidation, and supercomplex assemblies provide no kinetic advantage for quinone-mediated reactions.

In summary, Figure 2 demonstrates unambiguously that quinone/quinol freely exchange in and out of supercomplex assemblies to react with any enzyme in the membrane: they are not channeled between the enzymes in a single supercomplex. In fact, quinol is oxidized more rapidly by AOX external to the supercomplex assembly than by CIII/CIV internal to it. The following sections describe experiments that serve to confirm this result, by demonstrating that neither the supercomplexes nor the membrane integrity is compromised by AOX addition, and that AOX-catalyzed NADH oxidation is fully functional for oxidative phosphorylation.

### Supercomplex and Membrane Integrity Are Conserved Following AOX Addition

AOX purification requires a detergent, *n*-dodecyl β-D-maltoside (DDM), to maintain it in solution. Consequently, adding increasing amounts of AOX to membrane vesicles also adds increasing amounts of detergent, which could disrupt the supercomplex and/or membrane integrity.

**Figure 3:**
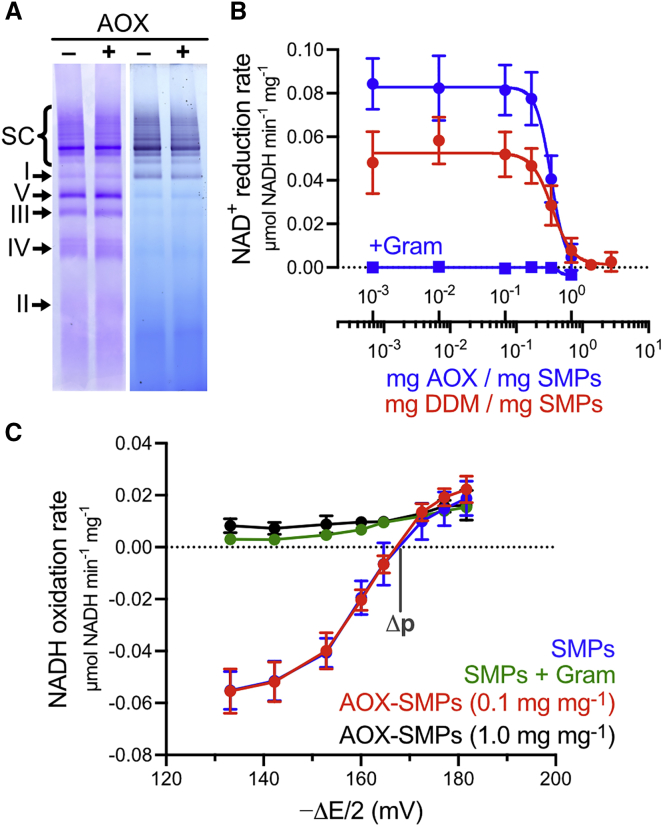
Supplementation with 0.1 mg AOX/mg SMPs Does Not Affect the Respiratory-Chain Supercomplexes, the Membrane Integrity, or Δ*p* (A) Blue native PAGE analysis of SMPs with and without 0.1 mg AOX mg^−1^ (10 μg SMPs per lane) stained with Coomassie R250 (left) or using an NADH oxidase activity stain (right). SC denotes the supercomplex bands. (B) Catalysis of the RET reaction (NAD^+^ reduction) by SMPs as a function of AOX (blue) and DDM (red) concentrations. Each point is a mean ± SD (n = 6). Blue squares show the uncoupling effect of adding 5 μg mL^−1^ gramicidin in the presence of different AOX concentrations (n = 3). (C) Dependence of the rate of NADH oxidation (or NAD^+^ reduction) on Δ*E*, modulated by varying the fumarate concentration against fixed NADH, succinate, and NAD^+^ concentrations (STAR Methods). SMPs (50 μg mL^−1^) were treated with 0.1 or 1.0 mg AOX mg^−1^ or 5 μg mL^−1^ gramicidin, as indicated. The data are presented as mean ± SD (SMPs, n = 11; SMPs + gramicidin, n = 3; AOX-SMPs [0.1 mg mg^−1^], n = 10; AOX-SMPs [1 mg mg^−1^], n = 3).

Figure 3A shows a blue native (BN)-PAGE analysis (Schägger and von Jagow, 1991, Wittig et al., 2007) of the supercomplexes present in control SMPs and those treated with 0.1 mg AOX mg^−1^, the ratio required for maximum catalysis. The supercomplex bands, visualized in the upper portion of the gel by either Coomassie or CI activity staining, match closely between the two samples, and also match the pattern observed in solubilized mitochondria (Blaza et al., 2014). The bands also exhibit the same apparent molecular weight, regardless of the presence of AOX. Therefore, supercomplexes retain their integrity in the presence of 0.1 mg AOX mg^−1^, and any kinetic effects observed cannot be ascribed to them being either augmented by AOX or disrupted by added detergent.

In reverse electron transfer (RET), NAD^+^ reduction by CI, linked to succinate oxidation by CII, is driven by high Δ*p* (Chance and Hollunger, 1960, Pryde and Hirst, 2011). Therefore, RET requires an intact, well-coupled membrane and it can be used to probe the membrane integrity. First, Figure 3B shows how the rate of RET (NAD^+^ reduction) catalyzed by SMPs drops precipitously above 0.25 mg AOX mg^−1^ or 0.17 mg DDM mg^−1^, well above the 0.1 mg AOX mg^−1^ required for maximal catalysis. Because the individual enzyme activities remain high, the results suggest catastrophic failure of the membrane. The concentrations of DDM and AOX at their apparent IC_50_ values of 0.35 mg DDM mg^−1^ (0.0018% DDM) and 0.5 mg AOX mg^−1^ and are 685 and 12.6 nmol mg^−1^, a ratio of 54:1 DDM/AOX. Using size-exclusion chromatography, ∼42 DDM molecules were estimated to be bound to each AOX monomer and a further 10 DDM molecules per AOX are free in the buffer used for the AOX stock solution, giving a total estimate of 52:1 DDM/AOX. Therefore, the membrane fails because of the DDM added along with the AOX, not because of AOX incorporation into the membrane. Second, the Δ*p* from ATP hydrolysis, used to drive the RET reaction, was determined in the presence and absence of AOX by varying the potential difference across CI and CII (Δ*E*) to identify the “balance” point of zero net flux where 4Δ*p* = −2Δ*E* (Pryde and Hirst, 2011). The assay does not involve AOX catalysis, so it was conducted in an anaerobic glovebox; ascofuranone could not be used to inhibit AOX because it also inhibits RET (but not NADH oxidation) by CI (IC_50_ = 0.44 ± 0.04 μM). Figure 3C shows that untreated SMPs and SMPs supplemented with 0.1 mg AOX mg^−1^ exhibit essentially the same Δ*p* (170.0 ± 0.2 and 167.8 ± 0.8 mV, respectively), matching reported values for bovine heart SMPs (∼160 mV) (Pryde and Hirst, 2011) and mitochondria (∼180 mV) (Nicholls and Ferguson, 2013). In contrast, SMPs supplemented with 10-fold more AOX are unable to sustain any substantial Δ*p*, similar to SMPs uncoupled by gramicidin. Together these results show that 0.1 mg AOX mg^−1^ does not substantially disrupt the membrane integrity.

### ATP Synthesis Driven by AOX-Catalyzed NADH Oxidation

**Figure 4:**
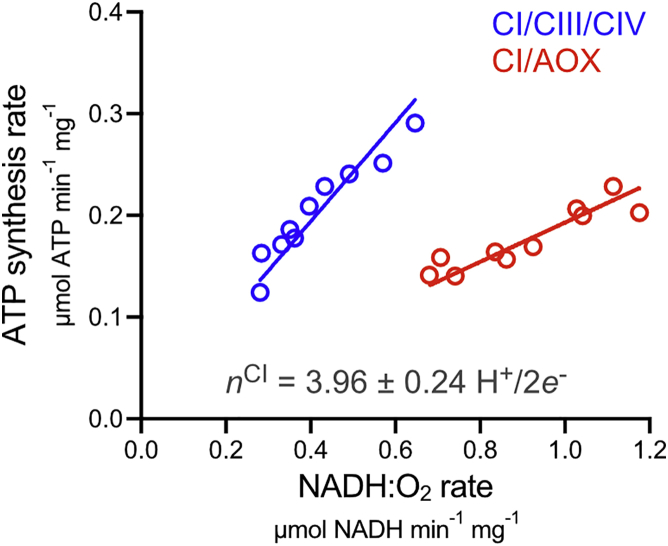
Addition of AOX to SMPs to Determine the H^+^/2*e*^–^ Stoichiometry of Complex I The rate of ATP synthesis driven by NADH oxidation through CI/CIII/CIV or CI/AOX is shown as a function of the rate of NADH oxidation. See STAR Methods for experimental details. The linear regression fits through the origin have slopes (±SE of the fit) of 0.485 ± 0.013 (r^2^ = 0.8663) and 0.193 ± 0.0046 (r^2^ = 0.8032) ATP NADH^−1^, respectively. The ratio of slopes equates to the CI stoichiometry (*n*^CI^) shown.

Finally, the proton-pumping stoichiometry of the CI/AOX respiratory chain was determined to confirm it is competent for oxidative phosphorylation. Recently, the 4H^+^/2*e*^–^ stoichiometry of CI was determined by comparing the rates of NADH:Q1, NADH:O_2_, and succinate:O_2_ oxidoreduction required to produce the same rates of ATP synthesis (Jones et al., 2017). Here, overlapping ranges of ATP synthesis rates were obtained for the CI/AOX and CI/CIII/CIV pathways, with ascofuranone (Minagawa et al., 1996) and cyanide used to turn off one pathway at once and a range of ADP-ribose concentrations used to adjust the rates by competitive inhibition of the CI flavin site (Zharova and Vinogradov, 1997). The rates of ATP synthesis depend linearly on the rates of NADH oxidation (Figure 4), and comparison of their gradients showed that 2.51 ± 0.09 times as much ATP per NADH is synthesized by the CI/CIII/CIV pathway compared to the CI/AOX pathway. Applying the established 6H^+^/2*e*^–^ stoichiometry for CIII/CIV catalysis (Nicholls and Ferguson, 2013) then yields a CI stoichiometry of 3.96 ± 0.24 H^+^/2*e*^–^, in excellent agreement with most previous studies (Galkin et al., 2006, Jones et al., 2017, Ripple et al., 2013, Wikström, 1984). Supplementing membrane vesicles by AOX may thus prove useful in future studies of CI H^+^/2*e*^–^ stoichiometries.

## Discussion

A functional role for respiratory-chain supercomplexes, to sequester quinone and thereby facilitate electron transfer between CI and CIII by quinone channeling, has been widely discussed (Bianchi et al., 2004, Lapuente-Brun et al., 2013, Lenaz et al., 2016, Schägger, 2000). Although recent biophysical and biochemical strategies (Blaza et al., 2014, Trouillard et al., 2011) do not support that supercomplexes confer a catalytic advantage in this way, the hypothesis survives because it provides a satisfying explanation for why supercomplexes exist, and because detailed biophysical experiments, based on time-resolved kinetic and thermodynamic approaches, lack accessibility to the wider community. Here, we have described a conceptually simple and straightforward experiment that demonstrates unambiguously that supercomplexes do not sequester their own individual quinone pools or benefit from enhanced catalysis due to quinone channeling.

A channeled substrate, by its nature, should be insensitive to competing enzymes. Here, we utilized a cyanide-insensitive, non-electrogenic quinol oxidase, AOX, as an enzyme that competes with CIII/CIV for quinol. If the quinol, generated by CI, is retained in the supercomplex, then AOX should have little impact on the rate of NADH oxidation or its sensitivity to cyanide. In contrast, we observed that, with increasing AOX supplementation, the rate of NADH oxidation increases substantially and becomes cyanide insensitive. The same behavior is observed for succinate oxidation by CII, which is not generally considered to be present in supercomplexes (Lobo-Jarne and Ugalde, 2018), and the fact that AOX-catalyzed NADH oxidation is actually faster than the CIII/CIV-catalyzed reaction underlines the lack of catalytic advantage from the supercomplex structure. The integrity of the supercomplexes, the membrane, and the oxidative phosphorylation process were all verified in the presence of AOX. Thus, our results confirm that quinol is not channeled or sequestered in supercomplexes, consistent with other rigorous biochemical and biophysical findings (Blaza et al., 2014, Kröger and Klingenberg, 1973, Kröger et al., 1973).

The following arguments are also against respiratory-chain supercomplexes sequestering or channeling ubiquinone/ubiquinol.

(1) In multi-enzyme complexes in which channeling has been demonstrated (Wheeldon et al., 2016), either there is a physical tunnel through which a substrate is shuttled, such as in tryptophan synthase (Dunn et al., 1990), or an intermediate is covalently tethered to a swinging arm, for example in the pyruvate dehydrogenase or fatty acid synthase complexes (Perham, 2000). In contrast, recent structures of the respirasome not only show minimal inter-protein contact between CI and CIII, but also that the quinone/quinol-binding sites are a substantial ∼100 Å distance apart and not oriented to facilitate channeling (Bauler et al., 2010, Gu et al., 2016, Guo et al., 2017, Letts et al., 2016).
(2) Electrostatically guided, bounded diffusion (Bauler et al., 2010, Wheeldon et al., 2016) occurs in the malate dehydrogenase/citrate synthase complex of the tricarboxylic acid cycle: the negatively charged oxaloacetate is channeled along a positively charged surface region linking the two active sites (Bulutoglu et al., 2016). In contrast, both quinone and quinol are neutral and extremely hydrophobic molecules, rendering them unsuitable substrates for this mechanism of channeling.
(3) The purpose of substrate channeling is typically to prevent a toxic intermediate from escaping into the cellular environment, or to drive up the local concentration of a substrate and push unfavorable equilibria forward (Wheeldon et al., 2016). Neither purpose is relevant here. Although quinol is able to react slowly with oxygen, it is a much lesser source of ROS generation in mitochondria than CI or CIII (Murphy, 2009), and also a natural antioxidant and signaling molecule in the cell (Crane, 2001).
(4) Electron transfer between CI and CIII is a diffusion-coupled, but not diffusion-controlled, phenomenon (i.e., quinone diffusion is faster than respiratory-chain turnover; Gupte et al., 1984). Increasing the rate of quinone transfer between enzymes would therefore provide no enhancement for the overall rate of respiratory-chain catalysis.

The major caveat of our experimental system is that our membranes are studied in non-physiological dilute solution, and thereby lack the mitochondrial environment and interacting pathways. However, *Ciona intestinalis* AOX has been expressed in *Drosophila* (Fernandez-Ayala et al., 2009), mice (El-Khoury et al., 2013, Szibor et al., 2017), and cultured human (HEK293-derived) cells (Hakkaart et al., 2006). In mice, BN-PAGE revealed that AOX does not affect the individual respiratory complexes or disrupt their organization into supercomplexes (El-Khoury et al., 2013, Szibor et al., 2017). Although AOX expression did not produce marked phenotypic effects in any case and had minimal impact on respiration rates in isolated mitochondria (presumably, quinol oxidation is not rate limiting), it conferred substantial cyanide and antimycin resistance on respiration. Therefore, the AOX is functional and, for it to be functional, quinol must be able to freely exchange in and out of the supercomplexes, consistent with the results of our study and our conclusion that quinone channeling is not required to support respiration. Consequently, both our biophysical work and these more physiologically relevant studies underline the potential of AOX to rescue CIII/CIV deficiencies through functional catalysis in mammalian cells (El-Khoury et al., 2013, Fernandez-Ayala et al., 2009, Mills et al., 2016).

Finally, if respiratory-chain catalysis does not depend on sequestration/channeling of substrates within supercomplex assemblies, why have they evolved? First, CI especially has been proposed to exhibit decreased ROS generation (Maranzana et al., 2013) and improved stability when it is in a supercomplex (Acín-Pérez et al., 2004, Diaz et al., 2006). Second, preferred weak interactions between the complexes may protect against non-specific aggregation in the high protein concentration of the mitochondrial inner membrane (Blaza et al., 2014). Third, supercomplex formation may promote quinone diffusion in the protein-dense membrane. Diffusion simulations of plastoquinone in grana thylakoid membranes (Tremmel et al., 2003), which also exhibit extremely high protein/lipid ratios, display higher quinone diffusion rates in simulations involving supercomplexes (large diffusion obstacles) than in simulations with individual (smaller) protein obstacles (Kirchhoff, 2014, Tremmel et al., 2003). Finally, supercomplexes may ensure a homogeneous distribution of complexes in the membrane, preventing segregation of the different complexes due to their different preferences for membrane topology (Cogliati et al., 2013), and thus prevent localized buildup and depletion of quinone and quinol.

## STAR★Methods

### Key Resources Table

**Table:** 

REAGENT or RESOURCE	SOURCE	IDENTIFIER
**Bacterial Strains**		

*Escherichia coli* FN102, *fhuA2* [*lon*] *ompT gal* (*λ DE3*) [*dcm*] *ΔhsdS*, *λ DE3 = λ sBamHIo ΔEcoRI-B int::*(*lacI::PlacUV5::T7 gene1*) *i21 Δnin5*, *ΔhemA*::*Km*^R^	Provided by Anthony Moore (University of Sussex). (Nihei et al., 2003)	N/A
*Escherichia coli* BL21(DE3), *fhuA2* [*lon*] *ompT gal* (*λ DE3*) [*dcm*] *ΔhsdS*, *λ DE3 = λ sBamHIo ΔEcoRI-B int::*(*lacI::PlacUV5::T7 gene1*) *i21 Δnin5*	New England Biolabs	C2527I

**Biological Samples**		

Bovine heart	C Humphreys & Sons Abbatoir, Chelmsford, UK	N/A

**Chemicals**, **Peptides**, **and Recombinant Proteins**		

Horse heart cytochrome *c* (≥95%)	Sigma-Aldrich	C2506
5-Aminolevulinic acid hydrochloride (≥98%)	Sigma-Aldrich	A3785
cOmplete Protease Inhibitor Cocktail	Roche	11836145001
Benzonase Endonuclease (>90%)	Merck	70746
DDM (≥99% α+β)	Anatrace	D310A
OG (≥99% α+β)	Anatrace	0311
d-Desthiobiotin (≥98%)	Sigma-Aldrich	D1411
NADH disodium salt hydrate (≥97%)	Sigma-Aldrich	N8129
NAD^+^ hydrate (≥98%)	Sigma-Aldrich	N1511
sodium succinate dibasic hexahydrate (≥99%)	Sigma-Aldrich	S2378
NADP^+^ sodium salt hydrate (≥98%)	Sigma-Aldrich	N0505
sodium fumarate dibasic (≥99%)	Sigma-Aldrich	F1506
Gramicidin	Sigma-Aldrich	G5002
Ascofuranone	Provided by Kiyoshi Kita (Nagasaki University)	N/A
NaCN (≥97%)	Sigma-Aldrich	71431
ADP-ribose sodium salt (≥93%)	Sigma-Aldrich	A0752
ADP sodium salt (≥95%)	Sigma-Aldrich	A2754
ATP disodium salt hydrate (≥99%)	Sigma-Aldrich	A7699
Digitonin, high purity - Calbiochem	Merck	300410
4x NativePAGE sample buffer	Invitrogen	BN2003
3-12% Bis-Tris NativePAGE gels	Invitrogen	BN1001BOX
Nitrotetrazolium blue chloride (>97%)	Sigma-Aldrich	N6639

**Critical Commercial Assays**		

Pierce BCA Protein Assay Kit	Thermo Scientific	23225
ATP Bioluminescence Assay Kit CLS II	Roche	11699695001

**Recombinant DNA**		

pET15b-N-terminal Twin-Strep AOX^Δ1-24^	(Fedor et al., 2017)	N/A
pASK40-*fumC*	Provided by Todd Weaver (University of Wisconsin-La Crosse) (Weaver et al., 1995)	N/A
pET-*maeB*	Provided by María Drincovich (National University of Rosario) (Bologna et al., 2007)	N/A

**Software and Algorithms**		

GraphPad Prism v7.0d	GraphPad Software	https://www.graphpad.com/scientific-software/prism/

**Other**		

Strep-Tactin Superflow high capacity resin	IBA	2-1208-025
Ni Sepharose 6 Fast Flow resin	GE Healthcare	17-5318-01

### Contact for Reagent and Resource Sharing

Further information and requests for resources and reagents should be directed to and will be fulfilled by the Lead Contact, Judy Hirst (jh@mrc-mbu.cam.ac.uk).

### Experimental Model and Subject Details

Bovine hearts from cattle of mixed gender, aged 18-22 months, were obtained from C Humphreys & Sons Abattoir, Chelmsford, U.K.

### Method Details

#### Preparation of Mitochondria

As previously detailed (Blaza et al., 2014), eight hearts were collected from freshly slaughtered cattle and immediately placed on ice. In a 4°C cold room, the hearts were trimmed of fat and connective tissue then minced in a meat grinder. One kilogram portions of mince were washed with 1.4 L of buffer A (250 mM sucrose and 10 mM Tris-HCl [pH 7.8 at 23°C]) then strained through a layer of muslin. Homogenisation was carried out with a Waring blender (30 s on high) with 1.6 L of buffer B (buffer A supplemented with 0.2 mM EDTA) and 25 mL 2 M Tris base. The homogenate was separated by centrifugation (2 600 g, 15 min, 4°C) and the pellet discarded. The muslin-filtered supernatant was centrifuged (13 000 g, 27 min, 4°C), then the pellets were retained, pooled and resuspended in ∼1.2 L buffer B. After pelleting the 3-4 aliquots (13 000 g, 42 min, 4°C), supernatants were discarded and the pellets were stored at −80°C.

#### Preparation of SMPs

SMPs were prepared from bovine heart mitochondria as described previously (Blaza et al., 2014, Pryde and Hirst, 2011). Mitochondria (∼10 g wet mass) were resuspended in 140 mL of SMP buffer (10 mM Tris-SO_4_ [pH 7.5 at 4°C], 250 mM sucrose) and centrifuged (11 300 g, 12 min, 4°C). The pellets were resuspended in a total of 30 mL SMP buffer, homogenized, then the pH was carefully adjusted to 9 with 2.5 M Tris base and the mixture left to incubate on ice for 10 min with stirring. The mitochondria were diluted to 140 mL with SMP buffer then collected by centrifugation (37 900 g, 14 min, 4°C), resuspended to 40 mL in SMP buffer and homogenized. Two further spin-resuspension cycles were conducted to wash the mitochondria. Unless indicated otherwise, the final resuspension was brought to 37 mL and 100 μM horse heart cytochrome *c* was added before the solution was left to incubate on ice for 1 h. MgSO_4_ was added slowly to 15 mM, then the mitochondria were probe sonicated on ice for ten 15 s bursts (150 W) with 1 min intervals. Aliquots of 5 mL were centrifuged (24 700 g, 20 min, 4°C) and the suspended SMPs were pelleted (74 700 g, 30 min, 4°C). They were then resuspended, homogenized and stored at 10-20 mg mL^-1^ at -80°C. Variability in the absolute rates of catalysis (but not the behavior) was observed between batches, reflecting variability in the starting material.

#### Preparation of AOX

AOX was prepared exactly as detailed previously (Fedor et al., 2017, Jones et al., 2016), based on the work of Kido and coworkers (Kido et al., 2010). AOX was overexpressed in a *hemA* deficient derivative of *Escherichia coli* BL21(DE3) (FN102) (Nihei et al., 2003) from the plasmid pET15b-*aox* (Nihei et al., 2003) carrying an N-terminal Twin-Strep tag (Schmidt et al., 2013) and with the first 24 residues (mitochondrial targeting sequence) deleted. 60 L of media [10 g L^-1^ tryptone, 5 g L^-1^ yeast extract, 5 g L^-1^ casamino acids, 10.4 g L^-1^ K_2_HPO_4_, 3 g L^-1^ KH_2_PO_4_, 0.74 g L^-1^ trisodium citrate dihydrate, 2.5 g L^-1^ (NH_4_)_2_SO_4_, 100 mg L^-1^ ampicillin, 50 mg L^-1^ kanamycin, 0.05 g L^-1^ MgSO_4_, 0.025 g L^-1^ FeSO_4_, 0.025 g L^-1^ FeCl_3_, 0.2% glucose, 3 mL antifoam 204] were inoculated in a fermenter to ∼0.01 OD_600_ from a 1 L pre-culture (further supplemented with 100 mg L^-1^ 5-aminolevulinic acid) and the culture grown at 30°C, 60% O_2_ saturation. Expression was induced with 25 μM IPTG when the OD_600_ reached ∼0.6 then the cells were harvested 12 h later.

Membranes were prepared immediately from the collected cells by disrupting them with two passes at 30 kPa through a Constant cell disruption system (Constant Systems Ltd) in the presence of 1 tablet per 50 mL^-1^ of cOmplete protease inhibitor (Roche), 2.5 U ml^-1^ benzonase, and 1 mM MgSO_4_ in 50 mM Tris-HCl [pH 7.5 at 23°C]. The membranes were then collected by centrifugation, resuspended to ∼30 mg mL^-1^ and frozen at −80°C in 20 mL aliquots. To prepare AOX, an aliquot of membranes was solubilised at 6 mg mL^-1^ with 1.4% OG, 25 mM Tris-HCl [pH 8.0 at 4°C], 200 mM MgSO_4_ and 20% (v/v) glycerol on ice for 1 h. AOX was purified with an 8 mL column of Strep-Tactin Superflow high capacity resin. After washing with strep buffer (20 mM Tris-HCl [pH 8.0 at 4°C], 50 mM MgSO_4_, 160 mM NaCl, 20% (v/v) glycerol, 0.042% DDM), AOX was eluted with strep buffer supplemented with 2.5 mM desthiobiotin. AOX-containing fractions were pooled and concentrated 10-fold, then dialyzed for 6 h against 2 L of strep buffer. Aliquots of AOX (3-5 mg mL^-1^) were stored at −80°C.

#### AOX Supplementation of SMPs

The incorporation of AOX into SMP membranes was typically conducted by incubating the desired quantity of AOX with 200 μg mL^−1^ of SMPs on ice in assay buffer for 15 to 60 min, although incorporation was found to be essentially instantaneous.

#### Preparation of FumC and MaeB

*Escherichia coli* fumarase (FumC) and oxaloacetate-decarboxylating malate dehydrogenase (MaeB) were overexpressed in BL21(DE3) cells transformed with the *maeB* or *fumC* plasmid, exactly as previously described (Bologna et al., 2007, Jones and Hirst, 2013, Weaver et al., 1995). Cultures were grown at 32°C in LB medium supplemented with 50 μg ml^-1^ ampicillin and induced at OD_600_ ∼0.6 with IPTG (1 mM for FumC and 0.1 mM for MaeB) for 18 h at 20°C. Cells were harvested by centrifugation (3 500 g, 10 min, 4°C), resuspended in buffer A (50 mM potassium phosphate [pH 7.8 at 4°C] and 300 mM NaCl) for FumC or buffer B (20 mM Tris-HCl [pH 7.4 at 4°C], 100 mM NaCl, 25 mM imidazole, and 10% (w/v) glycerol) for MaeB, then lysed using a Constant cell disruption system (Constant Systems Ltd) at 30 kPa. The lysates were centrifuged (150 000 g, 45 min, 4°C) then the supernatants were loaded onto pre-equilibrated 25 ml Ni-Sepharose 6 Fast Flow columns (GE Healthcare). The columns were washed using the respective buffers supplemented with 60 mM imidazole. FumC and MaeB were eluted with the appropriate buffer supplemented with 400 mM or 300 mM imidazole, respectively. Fractions were pooled, concentrated then dialyzed overnight in 10 mM Tris-SO_4_ [pH 7.0 at 4°C], 5 mM EDTA, and 1 mM dithiothreitol, for FumC and 60 mM Tris-SO_4_ [pH 7.4 at 4°C], 20 mM β-mercaptoethanol, 25 mM imidazole, and 10% (w/v) glycerol for MaeB.

#### Protein Quantification

Protein concentrations were determined by the bicinchoninic acid assay, following solubilization of SMPs with 2% SDS.

#### Activity Assays

All assays were conducted in a standard assay buffer (10 mM Tris-SO_4_ [pH 7.5 at 32°C] and 250 mM sucrose) at 32°C either in 1 mL cuvettes or in 96-well plates using a Molecular Devices SpectraMax Plus 384 microplatereader. NADH:O_2_ oxidoreduction was measured spectroscopically in 200 μM NADH (ɛ = 4.81 mM^-1^ cm^-1^ at 340 − 380 nm) (Birrell et al., 2009). Titration curves were measured in 96-well plates then the maximum rate for each curve was confirmed in cuvette experiments. Succinate:O_2_ oxidoreduction was determined indirectly by monitoring the NADPH (ɛ = 4.81 mM^-1^ cm^-1^ at 340 − 380 nm) generated by a coupled enzyme assay system composed of 5 mM succinate, 2 mM NADP^+^, 2 mM MgSO_4_, 1 mM K_2_SO_4_, 60 μg mL^-1^ FumC and 300 μg mL^-1^ MaeB (Jones and Hirst, 2013).

#### Blue Native PAGE

SMP samples were solubilized on ice at 2.5 mg mL^-1^, using 1% digitonin (high purity, Calbiochem) and 1x NativePAGE sample buffer (Invitrogen) for 20 min. Protein concentrations were determined following centrifugation at 14 000 *g* for 30 min at 4°C, then the samples were aliquoted and flash frozen for storage at −80°C. 10 μg of solubilized material was applied in each lane of 3-12% Bis-Tris NativePAGE gels (Invitrogen), run according to the manufacturer’s instructions. Proteins were visualized using Coomassie R250 staining or by an in-gel NADH oxidase activity assay using a 20 mM Tris-HCl [pH 7.0 at 23°C] solution of 100 μM NADH and 0.5 mg mL^−1^ nitrotetrazolium blue (Wittig et al., 2007).

#### RET Activity Assays and Measurement of Δp

Assays were conducted in an anaerobic glovebox at 32°C in assay buffer containing 1 mM NAD^+^, 10 mM succinate, 1 mM MgATP and 50 μg mL^-1^ SMPs. The MgATP was added to the SMPs before the other substrates and the NADH concentration monitored as described above. Δ*p* titrations were carried out using 100 μM NADH, 1 mM NAD^+^, 500 μM succinate, 1 mM MgATP and 50 μg mL^-1^ SMPs (prepared without additional cytochrome *c*). The concentration of fumarate was varied from 25 μM to 40 mM to set Δ*E* according to Equation 1 with Δ*E*_m,7.5_ = 0.33 V (Nicholls and Ferguson, 2013). Gramicidin (5 μg mL^-1^) was added from a DMSO stock solution so that the DMSO concentration did not exceed 0.5% (v/v). Δ*p* was determined from −Δ*E*/2 at the *x*-intercept of the data curve, as per Equation 2.

(Equation 1) $$\Delta E=\Delta E_{m,7.5}-\frac{RT}{2F}\ln\left(\frac{\left[Succ\right]\left[NAD^{+}\right]}{\left[Fum\right]\left[NADH\right]}\right)$$

(Equation 2) 4Δp=−2ΔE

#### ATP Synthesis and Measurement of CI Stoichiometry

The CI stoichiometry was determined using a method based on that of Jones and coworkers (Jones et al., 2017). SMPs supplemented with 0.02 mg AOX mg^-1^ were used to give appropriate rates of NADH oxidation and ATP synthesis. Ascofuranone (0.5 μM) and NaCN (0.4 mM) were used to inhibit the AOX or CIII/CIV pathways, respectively. ADP-ribose at 4, 6, 8 and 10 mM was used to give a range of NADH oxidation rates (Zharova and Vinogradov, 1997) for the CI/CIII/CIV pathway, and 0, 0.25, 0.5 and 1 mM ADP-ribose were used for the CI/AOX pathway. Linear regression through the origin of the rates of ATP synthesis versus the rates of NADH oxidation allowed for comparison of the slopes and calculation of the CI H^+^/2*e*^–^ stoichiometry. ATP concentrations were measured using the Roche ATP Bioluminescence assay kit CLS II in white 96-well plates using a Molecular Devices SpectraMax Gemini XPS microplate reader by comparison to known ATP/ADP standard solutions.

### Quantification and Statistical Analysis

The number of replicates, mean and standard deviation of measurements are reported in the relevant figure legends. Linear regressions and two-tailed t-tests were carried out using GraphPad Prism 7.
